# Spatial Patterns and Drivers of Microbial Taxa in a Karst Broadleaf Forest

**DOI:** 10.3389/fmicb.2018.01691

**Published:** 2018-07-26

**Authors:** Min Song, Wanxia Peng, Fuping Zeng, Hu Du, Qin Peng, Qingguo Xu, Li Chen, Fang Zhang

**Affiliations:** ^1^Key Laboratory of Agro-Ecological Processes in Subtropical Region, Institute of Subtropical Agriculture, Chinese Academy of Sciences, Changsha, China; ^2^Huanjiang Observation and Research Station for Karst Ecosystems, Institute of Subtropical Agriculture, Chinese Academy of Sciences, Changsha, China; ^3^Agricultural College, Hunan Agricultural University, Changsha, China; ^4^Key Laboratory of Land Surface Pattern and Simulation, Institute of Geographic Sciences and Natural Resources Research, Chinese Academy of Sciences, Beijing, China

**Keywords:** spatial pattern, driver, soil microbial communities, Illumina sequencing, karst forest

## Abstract

Spatial patterns and drivers of soil microbial communities have not yet been well documented. Here, we used geostatistical modeling and Illumina sequencing of 16S rRNA genes to explore how the main microbial taxa at the phyla level are spatially distributed in a 25-ha karst broadleaf forest in southwest China. *Proteobacteria*, dominated by *Alpha-* and *Deltaproteobacteria*, was the most abundant phylum (34.51%) in the karst forest soils. Other dominating phyla were *Actinobacteria* (30.73%), and *Acidobacteria* (12.24%). Soil microbial taxa showed spatial dependence with an autocorrelation range of 44.4–883.0 m, most of them within the scope of the study plots (500 m). An increasing trend was observed for *Alphaproteobacteria*, *Deltaproteobacteria*, and *Chloroflexi* from north to south in the study area, but an opposite trend for *Actinobacteria*, *Acidobacteira*, and *Firmicutes* was observed. *Thaumarchaeota*, *Bacteroidetes*, *Gemmatimonadetes*, and *Verrucomicrobia* had patchy patterns, *Nitrospirae* had a unimodal pattern, and *Latescibacteria* had an intermittent pattern with low and high value strips. Location, soil total phosphorus, elevation, and plant density were significantly correlated with main soil bacterial taxa in the karst forest. Moreover, the total variation in soil microbial communities better explained by spatial factors than environmental variables. Furthermore, a large part of variation (76.8%) was unexplained in the study. Therefore, our results suggested that dispersal limitation was the primary driver of spatial pattern of soil microbial taxa in broadleaved forest in karst areas, and other environmental variables (i.e., soil porosity and temperature) should be taken into consideration.

## Introduction

The spatial distribution of plants from small to large scales has been documented for a long period (Levin, 1992). Shifts in vegetation types, plant diversity, community, and biomass in terrestrial ecosystems can change the characteristics of soil nutrients and soil microbial communities (Batten et al., 2006; Murugan et al., 2014; Ren et al., 2018). These changes ultimately influence the functions of terrestrial ecosystems (Bell et al., 2005; Bardgett and van der Putten, 2014), which includes decomposition and biogeochemical nutrient cycling. A few famous ecological theories have highlighted the importance of resource quantity and the competition for resources in driving plant diversity and maintenance mechanisms in terrestrial ecosystems (Tilman, 1982; Hooper et al., 2000; Sterner and Elser, 2002). Meanwhile, plant diversity has been documented to be positively related with resource availability and heterogeneity, resulting in niche differentiation (Hooper et al., 2000; Sardans et al., 2012). Relative to the number of studies on the spatial distribution of plants, studies on the spatial patterns of soil microorganisms are recent and limited, although a growing number of studies have observed that microbes also exhibit spatial patterns at different scales (Horner-Devine et al., 2004; Martiny et al., 2006; Ranjard et al., 2010; Correa-Galeote et al., 2013).

Microorganisms are an integral part of terrestrial ecosystems and play a pivotal role in maintaining overall ecosystem functions (van der Heijden et al., 2008; Jing et al., 2015). The soil microbial community structure varies greatly in different spaces (Horner-Devine et al., 2004) and among different ecosystems. These variations are often explained by abiotic (e.g., soil properties) and biotic factors (e.g., plant functional traits) (de Vries et al., 2012). Among these factors, soil pH is regarded as a regulator of diversity in soil bacterial communities, such as *Acidobacteria* (Jones et al., 2009; Navarrete et al., 2015) at national and continental scales (Fierer and Jackson, 2006; Lauber et al., 2009; Griffith et al., 2011; Mukherjee et al., 2014). Nevertheless, great uncertainty remains whether soil pH itself is a direct factor or an indirect factor in shaping the spatial patterns of bacterial communities, which are influenced by plant traits, agricultural practices, soil nutrients, and many other factors. Therefore, the determinants or the underlying mechanisms of soil microbial community composition are not yet well understood due to a lack of studies at different spatial scales and locations, particularly in tropical and subtropical forests with high habitat heterogeneity, high plant biodiversity, and dynamic climates.

The spatial distribution of microbial communities and their determinants or underlying mechanisms has been investigated in many soil types, such as black soils (Liu et al., 2014), wetland (Ligi et al., 2014; Li et al., 2017), and Antarctic soils (Jung et al., 2011). However, similar studies in other special soil types (e.g., karst soil) have not been conducted due to high habitat heterogeneity and severe ecological degradation (Peng et al., 2008; Zhou et al., 2010; Jiang et al., 2014). Southwest China is one of the three largest karst areas in the world (Zhang et al., 2010). Karst is characterized by substantial soil erosion, widespread exposed bedrock, and poor soil carrying capacity (Peng et al., 2013), and the plants have specific adaptations, e.g., calcium-addiction, drought-tolerance, and lithophytes (Peng et al., 2008). Mixed evergreen and deciduous broadleaf forest is the representative vegetation cover in the karst region in southwest China, with complicated community structures, high biodiversity, and strong habitat heterogeneity (Zhang et al., 2010; Zhou et al., 2010; Du et al., 2015). However, the spatial distribution of soil microbial communities and its determinants remain unknown in the karst region.

In this study, we investigated the spatial distribution of the microbial communities in a 25-ha dynamic karst forest plot located in Mulun National Nature Reserve (MNNR), which is the largest forest reserve in the karst region and is a component of Chinese Forest Biodiversity Monitoring Network (CForBio) (Du et al., 2017). The microbial relative abundance in soil samples, collected using a grid method in the plot, was analyzed using Illumina Hiseq sequencing of 16S rRNA genes. The main objectives of this study were to (1) test whether there were spatial patterns of relative abundance of the microbial taxa at phylum level in the 25-ha karst forest plot; (2) generate maps of the patterns at the forest scale using geostatistical modeling, if spatial patterns existed; and (3) determine the drivers that influence the spatial patterns of soil microbial taxa in the karst forest. Our results can be used to forecast how soil microbial communities respond to changes in vegetation types in karst areas and to facilitate the sustainable management of karst ecosystems.

## Materials and Methods

### Study Area Description and Investigation of Plants and Topography

This study was conducted in the MNNR (107°54′ 01″- 108°05′51″E, 25°07′01″-25°12′ 22″N), which is in the northwestern region of Guangxi Province, China (Supplementary Figure S1). The MNNR is best preserved and largest primary karst forest, covering an area of 89.69 km^2^ with a range of 19.80 km from east to west and a distance 10.75 km from south to north. The subtropical monsoon climate has an average annual temperature of 19.38°C and average annual precipitation of 1,529.2 mm (mainly from April to August). In addition, the annual accumulated temperature above 10 is 6,260°C. The frost-free period is 310 days, and the mean annual relative humidity is 79% (Song et al., 2015).

A 25 ha (500 × 500 m) forest plot was established in MNNR in 2014, and subsequently the first census was conducted according to the standard field protocol of the Center for Tropical Forest Science (CTFS^1^). The plot was divided into a grid of 625 cells 20 × 20 m in size. All plant characteristics and topographical factors (elevation, slope, and slope aspect) were measured as previously described (Condit, 1998; Du et al., 2017), and plant diversity indices (richness index, Shannon index, and Simpson index) were determined as previously described in Green et al. (2005). Plant density was calculated as the number of the trees per unit area, soil depth was the mean depth of 8–10 points along an “S” shape in each cell, and rock outcrop coverage was the mean rock outcrop coverage of the corners and center of the cell. The spatial location of plot *i* is represented by *i*(X, Y), with the bottom left (southwestern) corner as the point of origin (0, 0), the Y axis running north and south, and the X axis running east and west (see in Supplementary Figure S2).

### Experimental Design and Soil Sampling

For soil microbial sampling, 25 20 × 20 m cells were taken as one large plot (100 × 100 m), and the samples were collected in the four corners and the center of each cell (Supplementary Figure S2). There are a total of 85 soil samples; however, during the process of transportation and analysis, 3 samples were contaminated and discarded. Therefore, there were 82 valid samples in the analysis.

Soil sampling was conducted in October 2016. After removing the litter, eight random samples (top 0–10 cm) were collected within a circle with a radius of 2 m (Supplementary Figure S3) using a soil auger with 5 cm inner diameter. The eight samples were homogenized into one composite sample per sampling point. The samples were immediately sent to lab and sieved through 2-mm mesh to remove rocks, roots, and debris. A portion of each sample was transported from the field to the laboratory in a liquid nitrogen tank and then stored at −80°C for DNA extraction. The remainder of the samples were air-dried and stored at air temperature prior to physical and chemical analysis.

### Soil Physicochemical Properties

Soil pH was determined using a pH meter after shaking the soil water (1:5 soil/water) suspension for 30 min. Soil organic carbon (SOC) and total nitrogen (TN) were determined using an elemental analyzer (Vario MACRO cube; Germany), total phosphorus (TP) was determined colorimetrically (UV, Spectrophotometer) after wet digestion with HClO_4_-H_2_SO_4_, and total potassium (TK) was determined by NaOH fusion-flame spectrophotometry. Available nitrogen (AN) was determined using the diffusion-absorption method, available phosphorus (AP) was determined by NaHCO_3_ extraction-ammonium molybdate spectrophotometry, and available potassium (AK) was determined by (NH_4_)_2_CO_3_ extraction-flame spectrophotometry (Bao, 2000).

### Soil DNA Extraction, PCR Amplification, and Sequencing of 16S rRNA Gene Data

Soil microbial DNA was extracted from each soil sample three times from 0.5 g of fresh soil (for a total of 1.5 g of soil) with the soil DNA kit (Fast DNA^®^SPIN Kit for Soil, MP), according to the manufacturer’s instructions. The concentration and quality of the extracted DNA were assessed with a spectrophotometer (NanoDrop2000, Thermo Scientific, Wilmington, DE, United States) and agarose gel electrophoresis. The extracted soil DNA was stored at −80°C for PCR amplification and 16S rRNA gene sequencing.

The V4–V5 region of 16S rRNA gene sequencing was targeted and amplified via PCR with the primers set 515F (5′-GTGCCAGCMGCCGCGGTAA-3′) and 907R (CCGTCAATTCCTTTG AGTTT-3′) (Biddle et al., 2008). The primer set provides comprehensive coverage with the highest taxonomical accuracy for microbial sequences (Mukherjee et al., 2014; Ren et al., 2016). The PCR reaction was performed in a 30 μl volume containing 15 μl of Phusion Master Mix 2x (Thermo Fisher Scientific Inc., Waltham, MA, United States), 3 μl of each primers (6 μM), 10 μl of DNA template (5–10 ng), and 2 μl H_2_O. After preparation, the samples denatured at 98°C for 1 min, then amplified using 30 cycles of 98°C for 10 s, 50°C for 30 s, and 72°C for 30 s, followed by extension at 72°C for 5 min. Each sample was amplified in three replicates. Finally, Illumina sequencing from each sample was conducted on an Illumina’s HiSeq 2000 platform (Illumina, San Diego, United States) at Novogene Biotechnology Co., Ltd. (Beijing, China).

The raw reads of microbial 16S rRNA genes were demultiplexed, quality-filtered, and processed by using QIIME based on three criteria (Ren et al., 2016). Sequence analysis was performed using the USEARCH v5.2.32 to filter and eliminate noise from the data by clustering similar sequences with <3% dissimilarity. Operational taxonomic units (OTUs) were clustered at the 97% similarity level using the UPARSE method (Edgar, 2010). Final OTUs were generated based on the clustering results, and taxonomic assignment was performed with the RDP 16S Classifier (Wang et al., 2007).

### Statistical Analyses

A geostatistical method was used to model the spatial structure of the relative abundance of the microbial taxa in the forest. First, the variables of relative abundance of the microbial taxa at the phylum level were analyzed using descriptive statistics. A Kolmogorov–Smirnov test revealed most of the measured variables followed a normal distribution. Four variables (i.e., *Chloroflexi*, *Nitrospirae*, *Latescibacteria*, and *Verrucomivrobia*) followed a normal distribution after using the Box-Cox transformation (Supplementary Table S1). Second, semivariogram models were calculated from GS+ 9.0 based on the transformed variables. Moran’s *I* index was used to measure whether a variable has a spatial dependency and whether the variable itself has a strong association in the nearest space (Fortin and Dale, 2005). Semivariance is a statistic measuring the spatial autocorrelation between samples at different lag distances:

(1) $$\gamma (h)=0.5*\frac{1}{N(h)}\sum {\left[Z_{i}-Z_{i+h}\right]}^{2}$$

Where *γ*(*h*) is semivariance for interval distance class *h*; *z_i_* is measured sample value at point *i*; *z_i+h_* is measured sample value at point *i*+*h*; and *N*(*h*) means total number of sample couples for the lag interval *h*. Either higher value of the higher determination coefficient (*R*^2^) or lower value of the residual sums of squares (RSS) of the best fitting model, which indicates that the spatial structure of soil microbial taxa at the phyla level can be better reflected (Supplementary Table S2). The distance at which the value of autocorrelation crossed the expected value was considered the ‘spatial range,’ which we termed ‘range’ for short in the study. The range indicated the maximal distance at which the variable is spatially autocorrelated (Fortin and Dale, 2005). Therefore, the range of main microbial taxa was within the sampling scale (<500 m), except for *Deltaproteobacteria* and *Firmicutes* (Supplementary Table S2), which suggested that the sampling scale was large enough to reveal spatial patterns of the main microbial taxa as well as the drivers of the spatial patterns. Third, ordinary kriging was used to make spatial prediction for points over the entire 500 × 500 m plot. The kriging maps were generated through ArcGIS 10.3 software.

Environmental factors included spatial position *i*(X, Y), soil properties (pH, SOC, TN, TP, TK, AN, AP, and AK), topographical variables (soil depth, rock outcrop coverage, elevation, slope, and slope aspect), and plant characteristics (richness index, Shannon index, Simpson index, and density). The relationships between spatial position, soil properties, topographical factors, plant characteristics, and microbial phyla were determined by Pearson’s correlation analysis in SPSS 18.0 software package. In order to decrease the false discovery rate, Benjamini-Hochberg procedure were conducted for multiple testing corrections. The relationship between soil bacterial phyla and the above mentioned environmental factors were identified using redundancy analysis (RDA) and conducted using Canoco 5.0 software. Before the RDA analysis, a detrended correspondence analysis (DCA) for the relative abundance of the main microbial taxa was performed to confirm that the linear ordination method was suitable for analysis of the microbial taxa data (gradient length <3). The result of RDA analysis revealed the effect of the environmental factors on microbial communities based on the arrow length and the angle between lines (ter Braak and Smilauer, 2002). Furthermore, the respective effects of spatial and environmental variables were determined by canonical variation partitioning (Borcard et al., 1992; Legendre et al., 2009). Partial RDA was performed in CANOCO 5.0.

## Results

### Plant Characteristics and Soil Physical Properties in MNNR

According to the first census in 2014, there were 144,552 individuals belonging to 51 families, 127 genera, and 228 species in the forest plot. The most dominant species were *Crytocarya microcarpa*, *Itoa orientalis*, *Platycarya longipes*, and *Lindera communis* (Du et al., 2017). In the microbial sampling plots, the mean richness index for woody plants was 30.5, the mean Shannon–Wiener index was 2.30, and the mean Simpson index was 0.74, and the mean plant density was 242 plants per plot (**Table 1**). Soil pH and SOC varied from 6.35 to 8.25 and 28.96 to 129.39 g kg^−1^, respectively. TN, TP, TK, AN, AP, and AK ranged from 3.00 to 14.41 g kg^−1^, 0.35 to 3.39 g kg^−1^, 1.05 to 13.96 g kg^−1^, 166.71 to 860.20 mg kg^−1^, 0.95 to 18.54 mg kg^−1^, and 1.23 to 12.91 mg kg^−1^, respectively, and had a medium variation with CV (Coefficient of variation) between 25 and 75%, except for pH (**Table 1**).

**Table 1:** Plant characteristics and soil properties in Mulun National Natural Reserve.

	Minimum	Maximum	Means	SE	CV (%)	Skewness	Kurtosis
***Plant characteristics***							
*Richness index* (*R*)	3	65	30.50	17.33	56.81	0.338	−0.996
Shannon index	0.10	3.60	2.30	1.04	45.26	−0.507	−1.097
Simpson index	0.03	0.96	0.74	0.27	35.92	−1.052	−0.237
Density (plants⋅plot^−1^)	53	735	242	130.9	54.09	1.613	3.459
***Basic soil properties***							
pH	6.35	8.25	7.45	0.47	6.32	−0.572	−0.665
SOC (g kg^−1^)	28.96	129.39	62.16	21.58	34.72	1.194	1.290
TN (g kg^−1^)	3.00	14.41	7.38	2.62	35.46	0.999	0.438
TP (g kg^−1^)	0.25	3.39	1.54	0.74	48.26	0.042	−0.803
TK (g kg^−1^)	1.05	13.96	5.33	2.93	54.93	0.615	−0.271
AN (mg kg^−1^)	166.71	860.20	423.84	157.56	37.18	0.736	−0.050
AP (mg kg^−1^)	0.95	18.54	5.43	3.58	65.89	1.294	1.611
AK (mg kg^−1^)	1.23	12.91	4.82	2.50	51.91	0.797	0.293

*SOC, soil organic carbon; TN, total nitrogen; TP, total phosphorus; TK, total potassium; AN, available nitrogen; AP, available phosphorus; AK, available potassium.*

### Soil Microbial Community Structure and Composition

**FIGURE 1:**
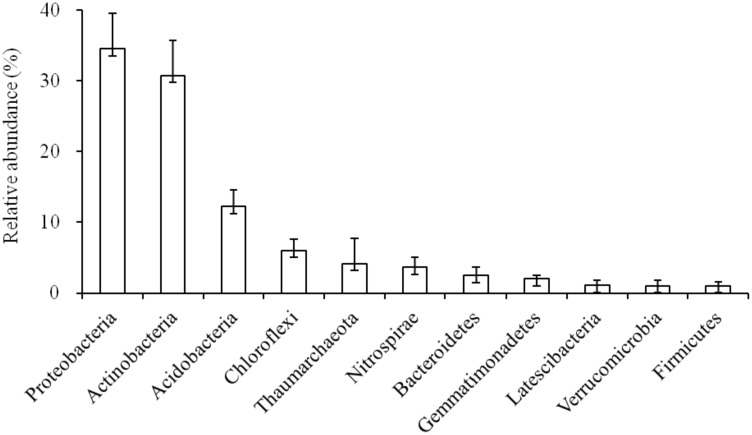
Soil bacterial community composition in the karst forest.

The relative abundance (>1%) of soil microbial phyla were: *Proteobacteria* (34.51%), *Actinobacteria* (30.73%), *Acidobacteria* (12.24%), *Chloroflexi* (6.00%), *Thaumarchaeota* (4.17%), *Nitrospirae* (3.61%), *Bacteroidetes* (2.45%), *Gemmatimonadetes* (2.00%), *Latescibacteria* (1.06%), *Verrucomicrobia* (1.04%), and *Firmicutes* (1.04%) (**Figure 1**, Supplementary Table S1). Notably, among these sequences, *Proteobacteria* was the most dominant phyla in the karst forest (**Figure 1**), which has two dominant *Proteobacteria* classes, *Alphaproteobacteria* and *Deltaproteobacteria*. Thus, *Alpha-* and *Deltaproteobacteria* were included in our analysis. At the class level, *Alphaproteobacteria* (18.43%) and *Thermoleophilia* (11.57%) were the most dominant classes (Supplementary Figure S4). Within *Alphaproteobacteria*, the orders *Rhizobiales* and *Rhodospirillales* were the most abundant in the soil. Within *Thermoleophilia*, the orders *Gaiellales* and *Solirubrobacterales* were dominate (Supplementary Figure S4). The CV of *Actinobacteria*, *Alphaproteobacteria*, and *Acidobacteria* were below 25%, whereas *Thaumaechaeota* and *Verrucomicrobia* were above 75%. Other phyla were between 25 and 75% (Supplementary Table S1).

### Spatial Autocorrelation of Microbial Communities

**FIGURE 2:**
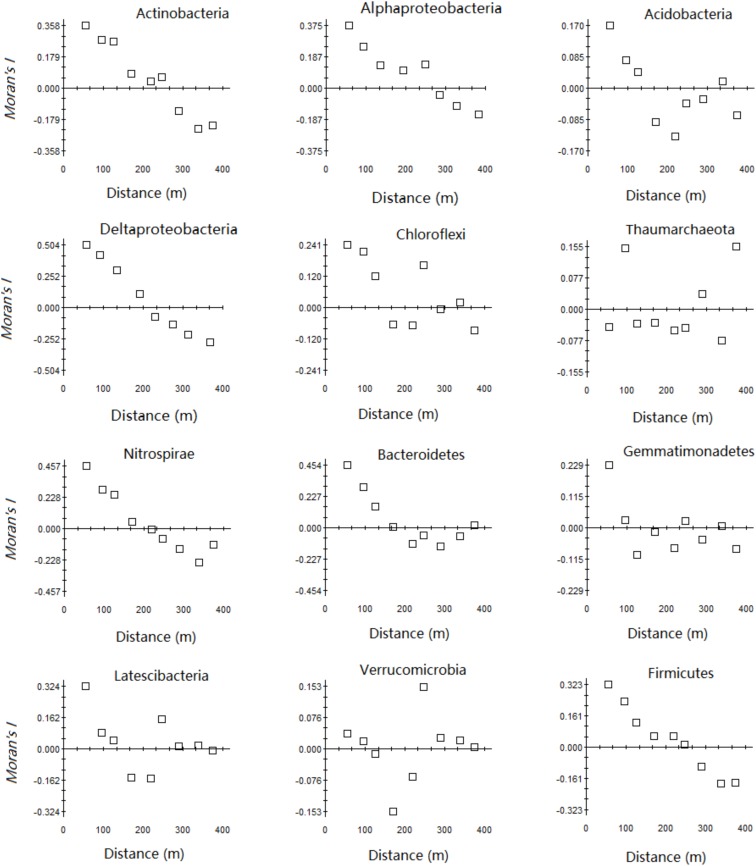
Moran’s *I* index of relative abundance of the main phyla in the karst forest.

There were three trends in the spatial autocorrelation of the relative abundance of the main microbial phyla in the karst forest (**Figure 2**). First, the spatial dependence of *Thaumarchaeota* was very small, the Moran’s *I* index was close to 0, and the regularity was not strong. The other ten phyla had a certain degree of spatial dependency, ranging from −0.157 to 0.495, and with a decreasing order of *Bacteroidetes*, *Nitrospirae*, *Proteobacteria*, *Actinobacteria*, *Latescibacteria*, *Firmicutes*, *Chloroflexi*, *Gemmatimona*, *Acidobacteria*, and *Verrucomicrobia*. Secondly, *Proteobacteria*, *Nitrospirae*, and *Firmicutes* had a similar spatial structure in that the Moran’s *I* index of these phyla gradually declined as the lag distance increased and reached 0 around 226.27 m. Then, the Moran’s *I* index of the other phyla decreased to negative and then increased after a minimum as the lag distance was extended, which illustrated a distinct spatial structure.

**FIGURE 3:**
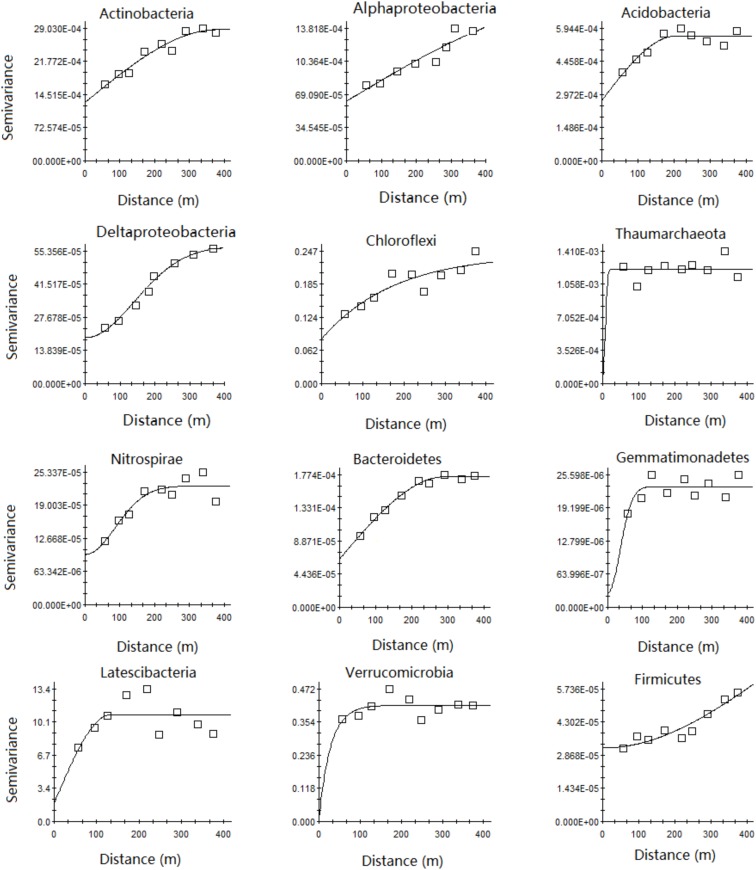
Semivariograms of relative abundance of the main microbial phyla. Semivariance models and parameters for all the studied phyla are given in Supplementary Table S3.

The Gaussian model fit best for *Alpha*- and *Deltaproteobacteria*, an exponential model fit best for *Thaumarchaeota*, *Nitrospirae*, *Verrucomocrobia*, and *Firmicutes*, and a spherical model fit best for the other bacterial phyla (Supplementary Table S2 and **Figure 3**). Geostatistical modeling showed a very low nugget effect (*C*_0_) and autocorrelation range of 44.4–883.0 m for the main microbial phyla relative abundance in the karst forest soil (Supplementary Table S2 and **Figure 3**). The range of most of the main microbial taxa (except *Deltaproteobacteria* and *Firmicutes*) was less than the sampling range (500 m). Moreover, the range of *Thaumarchaeota* and *Verrucomicrobia* was very small, 44.4 and 84.3 m, respectively (Supplementary Table S2).

**FIGURE 4:**
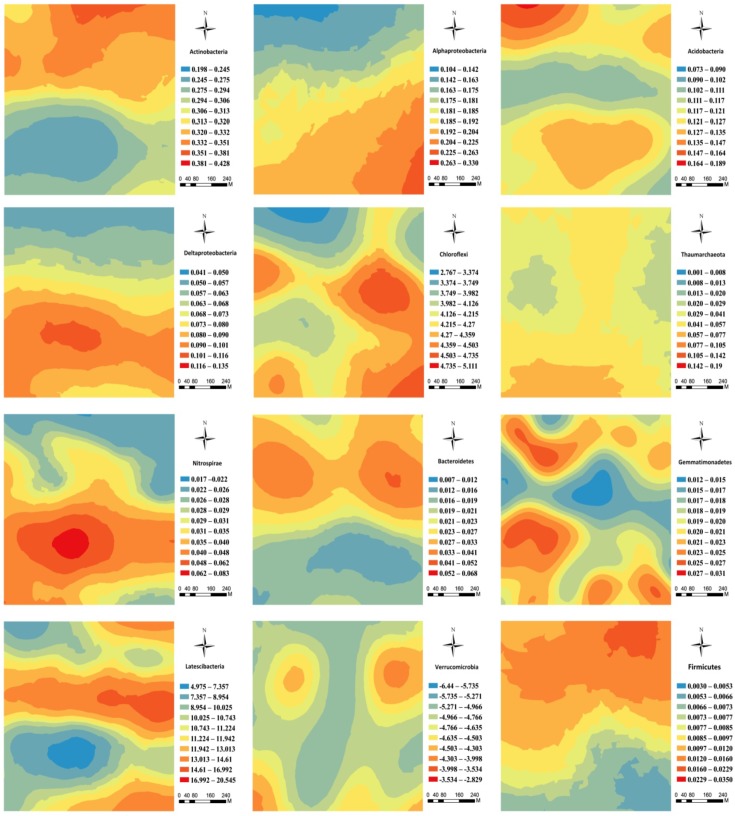
Spatial distribution of the main soil microbial phyla in the karst forest.

The kriging maps showed an increasing trend in the spatial distribution of *Alphaproteobacteria*, *Deltaproteobacteria*, and *Chloroflexi* from the north to the south of the forest field (**Figure 4**), but *Actinobacteria*, *Acidobacteira*, and *Firmicutes* showed an opposite trend (**Figure 4**). Moreover, *Thaumarchaeota*, *Bacteroidetes*, *Gemmatimonadetes*, and *Verrucomicrobia* had a distinct patchy pattern (**Figure 4**). *Nitrospirae* displayed a unimodal distribution pattern with the peak value of relative abundance in the middle part of the plot (**Figure 4**). By and large, *Latescibacteria* exhibited an intermittent pattern with high and low value strips (**Figure 4**).

### Relationship Between Soil Microbial Taxa and Environmental Factors

Pearson’s correlation analysis showed a significant correlation between *Y* values of the plot locations, phosphorus, elevation, slope, plant density, plant diversity (i.e., richness index, Shannon index, and Simpson index), and the relative abundance of many soil microbial taxa (**Table 2**). Dynamics in soil microbial community composition, induced by relative abundance, were most closely related to the plot location, phosphorus, and plant factors. However, microbial community composition was unrelated to SOC, AK, soil depth, rock outcrop coverage, and slope aspect. We also found that plant traits and topographical factors were highly correlated with X value, TK and TP were highly correlated, tree abundance and Simpson index were highly correlated with Shannon index, soil depth and rock outcrop coverage were highly correlated, and slope and elevation were also highly correlated (Supplementary Table S3). Thus, these correlated variables were removed before RDA analysis. According to the result of forward selection, the variables including pH, AN, Rockcov, TN, AK, SOC, and Aspect, were removed in the RDA analysis.

**Table 2:** Pearson’s correlation coefficients between the main phyla and environmental factors (*n* = 82).

	Actinob	Alphap	Acidob	Deltap	Chlorof	Thaumar	Nitrosp	Bactero	Gemmati	Latesci	Verruco	Firmicu
Y	**0.475^∗^**	**−0.590^∗^**	0.161	**−0.615^∗^**	**0.469^∗^**	**−**0.171	**−0.408^∗^**	**0.421^∗^**	**−**0.131	**−**0.108	**−**0.167	**0.523^∗^**
TN	**−0.055**	−0.134	**0.349^∗^**	**−**0.162	0.170	0.040	**−**0.108	0.064	0.058	**−**0.017	**−**0.116	0.064
TP	**−0.272**	−0.174	0.257	0.117	0.226	0.150	0.226	**−**0.184	0.251	**0.331^∗^**	**−**0.192	0.030
AP	**−0.141**	0.019	0.183	0.221	0.064	**−**0.030	0.101	**−**0.293	**0.366^∗^**	0.176	**−**0.048	**−**0.256
S	0.244	0.098	0.025	**−**0.245	**−**0.121	**−**0.043	**−**0.284	0.128	**−**0.286	**−0.343^∗^**	0.103	**−**0.028
Shannon	0.221	0.116	0.129	**−**0.259	**−**0.069	**−**0.043	**−0.329^∗^**	0.075	**−**0.301	**−0.369^∗^**	0.105	**−**0.106
Simpson	0.229	0.120	0.109	**−**0.285	**−**0.081	**−**0.048	**−0.369^∗^**	0.117	**−**0.309	**−0.424^∗^**	0.120	**−**0.113
Den	0.283	0.091	**−0.315^∗^**	**−**0.271	**−**0.208	**−**0.017	**−**0.233	0.243	**−**0.085	**−0.306^∗^**	0.082	0.086
Elev	0.139	**0.314**^∗^	−0.120	−0.125	**−**0.213	**−**0.208	**−**0.276	0.182	**−**0.132	−0.295	0.266	**−**0.034
Slope	0.166	0.211	−0.064	**−**0.218	**−**0.239	0.048	**−0.321^∗^**	0.148	**−**0.304	**−0.391^∗^**	0.063	**−**0.062

*^∗^indicates that correlations are significant at Q-value < 0.01 after p-value adjusting by Benjamini-Hochberg procedure. Data of the variables with weak relationships with soil microbial taxa are not shown. These are the abbreviation of microbial taxa: Actinobacteria (Actinob), Alphaproteobacteria (Alphap), Acidobacteria (Acidoba), Deltaproteobacteria (Deltap), Chloroflexi (Chlorof), Thaumarchaeota (Thaumar), Nitrospirae (Nitrosp), Bacteroidetes (Bactero), Gemmatimonadetes (Gemmati), Latescibacteria (Latesci), Verrucomicrobia (Verruco), and Firmicutes (Frimicu); the abbreviation of environmental characteristics: total nitrogen (TN), total phosphorus (TP), available phosphorus (AP); Y represent the location of the plots; elevation (Elev); Richness index (S), Shannon index (Shannon), Simpson index (Simpson), plant density (Den).*

**FIGURE 5:**
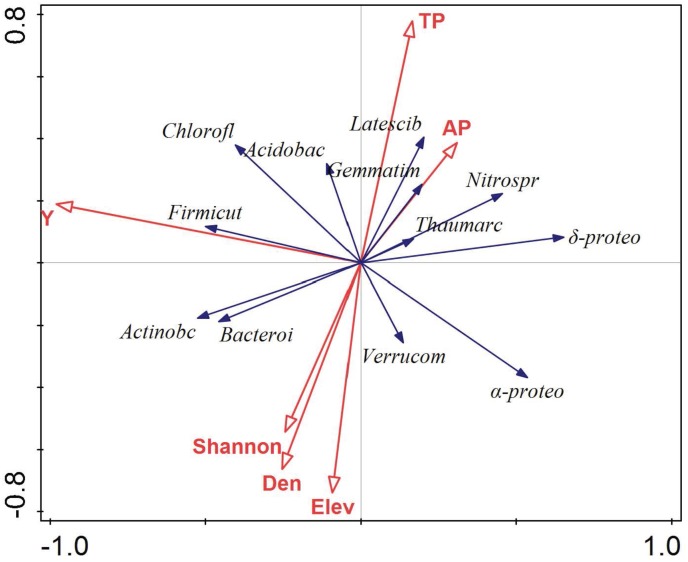
Ordination plots of the results from the redundancy analysis (RDA) to identify the relationships among the microbial taxa (blue arrows) and environmental factors (red arrows). These are the abbreviation of microbial taxa: *Actinobacteria* (*Actinobc*), *Alphaproteobacteria* (α*-proteo*), *Acidobacteria* (*Acidobac*), *Deltaproteobacteria (*δ*-proteo*), *Chloroflexi* (*Chlorofl*), *Thaumarchaeota* (*Thaumarc*), *Nitrospirae* (*Nitrospr*), *Bacteroidetes* (*Bacteroi*), *Gemmatimonadetes* (*Gemmatim*), *Latescibacteria* (*Latescib*), *Verrucomicrobia* (*Verrucom*), and *Firmicutes* (*Frimicut*); the abbreviation of environmental factors: the location of the plots (Y), total phosphorus (TP), available phosphorus (AP); elevation (Elev), Shannon index (Shannon), plant density (Den). Each vector points to the direction of increase for a given microbial phylum and its length indicates the strength of the correlation between the variable and the ordination scores.

**FIGURE 6:**
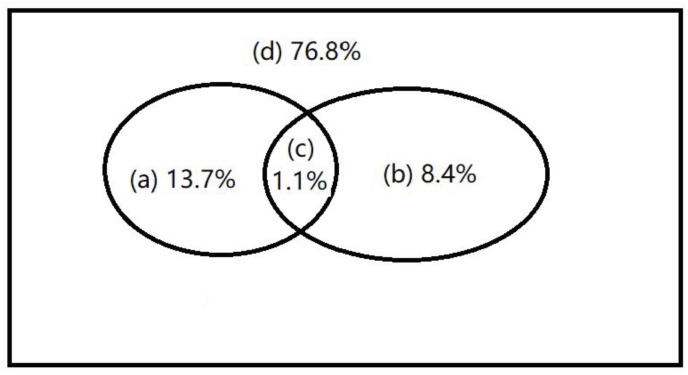
Variation partitioning of the main microbial taxa relative abundance. **(A)** spatial variation that is not shared by the environmental variables; **(B)** spatially structured environmental variation; **(C)** non-spatial environmental variation; and **(D)** unexplained variation and stochastic fluctuations.

The relationship between environmental variables (i.e., Y, TP, AP, Elev, Shannon, and Den) and the dominant microbial taxa relative abundance were examined using RDA (**Figure 5**). The results showed that environmental variables, especially Y value of location, TP, elevation, and plant density, significantly affected the soil dominant microbial community. Furthermore, the relative abundance of *Firmicutes*, *Actinobacteria*, *Bacteroidetes*, and *Chloroflexi* was positively correlated with Y value of location, while *Alpha-* and *Deltaproteobacteria* showed the opposite trend. *Latescibacteria*, *Gemmatimonadetes*, *Acidobacteria*, *Verrucomicrobia*, and *Bacteroidetes* were significantly affected by soil TP, elevation, and plant density. Variance partitioning indicated that environmental factors explained 8.4% of variation in spatial distribution of soil microbial taxa relative abundance, and spatial factors explained 13.7% of the variation. The interaction between environmental factors and spatial factors was 1.1% of the variation (**Figure 6**).

## Discussion

### Soil Microbial Community Structure and Composition

In the study, there were 11 phyla with >1% relative abundance in the karst forest soils, i.e., *Proteobacteria*, *Actinobacteria*, *Acidobacteria*, *Chloroflexi*, *Thaumarchaeota*, *Nitrospirae*, *Bacteroidetes*, *Gemmatimonadetes*, *Latescibacteria*, *Verrucomicrobia*, and *Firmicutes* (**Figure 2** and Supplementary Table S1). Moreover, about 77% of the obtained sequences in the karst forest soils belonged to three dominant phyla, i.e., *Proteobacteria* (34.51%), *Actinobacteria* (30.73%), and *Acidobacteria* (12.24%) (**Figure 1** and Supplementary Figure S4). The contribution of the dominant phyla was similar to the studies in black soils (78.0% of nine dominant groups; Liu et al., 2014), in Chinese boreal forest soils (76% of four dominant phyla; Xiang et al., 2014), and in Oklahoma prairie soil (75% of the three dominant phyla; Sheik et al., 2011), but higher than the studies in Loess afforested soils (69.9% of three dominant groups; Ren et al., 2016) and in wetland soils (65% of six dominant groups; Ligi et al., 2014), and lower than in Arctic soils (83.0% of five dominant groups; Chu et al., 2010).

It should be noted that the samples involved in the studied by Liu et al. (2014) and Chu et al. (2010) were obtained from agricultural and tundra soils, respectively. The most dominant phyla had strong correlations with soil pH in arctic soils (Chu et al., 2010), while no significant correlations were observed for *Alphaproteobacteria* in soils from lower latitude biomes (Lauber et al., 2009). Although the largest difference in measured soil pH was only 2.01 pH units, the large differences in soil C and N content in black soils, soil microbial community composition, phylotyope richness, and phylogentic diversity were significantly correlated with soil pH (Liu et al., 2014). The samples in the study by Ren et al. (2016) were collected from afforested soils, and soil N fractions, especially dissolved organic nitrogen (DON), were significantly correlated with most microbial groups and microbial diversity. Therefore, studies that compare the microbial communities between different soils types (e.g., black soil, wetland soil, and karst soil) or among different land use types (e.g., agriculture, tundra, prairie, and forest) need to be carried out in the future.

### Spatial Variations of Microbial Taxa

The relative abundances of the main microbial taxa at the phylum level were not randomly distributed at the 25-ha karst forest plot and could be mapped using geostatistical modeling. Our results confirmed that most microbial processes in soil have spatial variation at the local scale (Parkin, 1993). The results of Moran’s *I* index indicated both *Deltaproteobacteria* and *Firmicutes* showed autocorrelation patterns over larger scales (883.0 and 710.9 m, respectively) compared with other microbial phyla (ranging from 44.4 to 412.8 m) (**Figure 2** and Supplementary Table S2). Therefore, due to the scale considered in our study, almost no patchiness was observed for *Deltaproteobacteria* or *Firmicutes*, whereas smaller scale patchiness was found for other phyla. To better carry out spatial analysis of these two phyla, it is necessary to moderately reduce the scale or to increase sampling density to avoid missing related important information.

A very low nugget effect for most microbial communities in the study (Supplementary Table S2 and **Figure 3**) was also observed by Philippot et al. (2009) and Ritz et al. (2004) for other microbial properties. Although there is little literature about sampling at a similar spatial scale (500 m), the spatial dependence of soil microbial properties and taxa at different scales has been reported. For example, at the meter scale, extreme spatial variations in community-level microbiological properties and N-cycling microbial communities existed in upland grasslands (Ritz et al., 2004) and constructed wetland sediments (Correa-Galeote et al., 2013), respectively. The total and relative abundance of eight bacterial taxa displayed strong spatial distributions at the pasture scale (39.6 × 14.4 m), with varying or even contrasting patterns. For example, the relative abundance of both Acidobacteria and Verrumcomicrobia was lower in the central field, while the opposite trend was observed for Bacteroidetes (Philippot et al., 2009). Community components for both ammonia-oxidizing bacteria (AOB) and archaea (AOA) exhibited spatial patterns at the hectare scale (Wessén et al., 2011). At the landscape scale, the abundance of total bacteria, crenarchaea, nitrate reducers, denitrifiers- and ammonia oxidizers exhibited spatial distribution across 31,500 km^2^ (Bru et al., 2011). Thus, based on these studies, we deduced that the distribution of soil microbial taxa relative abundance in the karst forest was also not random on the meter scale and can be modeled at a forest scale.

### Correlation Between Soil Microbial Taxa and Environmental Factors

In our study, *i*(X, Y) represented the location of sample plot *i* by grid method from the origin. Y value showed to account most for soil microbial community composition in the karst forest among the selected environmental factors (**Figure 5**). It is well confirmed that soil properties, including soil microbial taxa, are spatially differentiated by both internal factors (natural conditions), which accounted for causing a strong spatial variability of soil properties, and by external factors (field management), which are thought to be responsible for the weak spatial dependence of soil properties (Atreya et al., 2008).

Soil TP was significantly correlated with main bacterial taxa in the karst forest (**Figure 5**), which was responsible much for the distribution of microbial community composition. Soil phosphorus, most of which was obtained from the parent material, was gradually depleted and fixed in plants and animal tissues and its concentrations became unsatisfactory for microorganisms (McGill and Cole, 1981), which can restrict the development of microbial community (Jangid et al., 2014). Moreover, the growth-rate hypothesis (Sterner and Elser, 2002) suggested that microorganisms belonging to fast-growing organisms require high P demand for the synthesis of ribosomes, ATP, DNA, and RNA (Peñuelas and Sardans, 2009). Therefore, to a large extent, P supply should be an important factor for microbial community composition and distribution in the karst forest soils.

Topographical factors, such as elevation and slope, were significantly negative correlated with most soil microbial taxa across the forest field (**Figure 5**). On one hand, this is because fine particles eroding from higher elevation and then deposing at the lower areas of the field where relatively fine soil offers more affordable living environment for microbes (Constancias et al., 2015). On the other hand, this topographical difference in soil microbial taxa resulted from the soil hydrological condition regulating soil carbon flux (Dai et al., 2012; Song et al., 2017), which affects soil aeration and influences soil microbial respiration (Dai et al., 2012). Soil depth and rock outcrop coverage slightly affected soil microbial taxa, even though shallow soil and high rock outcrop coverage is characteristic of karst (Song et al., 2015). This weak relationship might be because soil microorganisms are adapted to survive on the soil surface, appearing as colonies on the surface of pore walls.

Plant density had more effect on the soil microbial community composition in the karst forest than plant diversity indices (**Figure 5**). Higher plant density means more fine roots and a competition for soil nutrient in the soil. Root exudation extremely affects the availability of soil organic C and influences soil nutrient status (Phillips et al., 2011), which ultimately affects microbial niches, diversity, and activity (Sinsabaugh et al., 2008; Lopez-Sangil et al., 2011). Moreover, it has been observed that karst forests with more fine root biomass are favorable for soil microbial growth via decomposition and mineralization (Krashevska et al., 2015; Lai et al., 2016) as in afforested soils (Ren et al., 2016). In parallel, the relative abundance of *Nitrospirae*, *Gemmatimonadetes*, and *Latescibacteri* was lower and *Actinobacteria* was higher with increased plant diversity (**Figure 5** and **Table 2**), indicating that plant diversity can drive the changes in microbial diversity (Meier and Bowman, 2008; de Graaff et al., 2010).

Soil pH has been documented as the major factor determining the soil microbial community composition in many soils and ecosystems, such as soils across North and South America (Lauber et al., 2009), British soils (Griffith et al., 2011), Antarctic soils (Jung et al., 2011), black soils (Liu et al., 2014), wetlands (Ligi et al., 2014; Li et al., 2017), and a karst cave ecosystem (Yun et al., 2016). It is worthy to note the samples in these studies were collected from different soil types and the soil pH was highly variable, or that the samples were collected from one soil type with a large variation in pH and slight variation in other soil properties (such as soil C and N content). However, in our study, the largest difference in soil pH was only 1.90 pH units (seen in **Table 1**), and we found a low correlation between pH and the main microbial phyla relative abundance (Data not shown). Thus, our results indicated that soil pH did not play an important role in shaping microbial community composition in the karst forest soil. In addition, Fierer et al. (2007) found that the C mineralization rate was the best predictor of relative abundance of bacterial phyla. The soil C mineralization rate was not measured in our study, but we did find that SOC content had a low correlation with soil microbial phyla (Data not shown). This observation may be partially explained by relative abundance of microbial taxa. It was hardly affected by a fluctuation in the amount of starting material (e.g., SOC) between samples and thus did not depend on the exact quantification of DNA extracted from the soil and its inherent bias (Philippot et al., 2009).

### Drivers of Spatial Patterns of Microbial Taxa

The spatial distribution of soil microbial communities of the karst forest was heterogeneous and complex. The kriging maps showed that the microbial communities displayed different or even contrary spatial patterns, suggesting that they have different responses to spatial abiotic or biotic factors. For example, *Alphaproteobacteria, Deltaproteobacteria*, and *Chloroflexi* had an increasing trend from the north to the south in the forest plot, whereas the opposite was found for *Actinobacteria*, *Acidobacteira*, and *Firmicutes* (**Figure 4**). Overall, these six phyla could be differentiated into copiotrophic or oligotrophic categories. Our results indicated that the spatial heterogeneity of soil nutrients affect the r- and K-selected categories of microbial taxa (Fierer et al., 2007). In addition, the spatial pattern of *Actinobacteria* and *Acidobacteira* were roughly identical (**Figure 4**), but the trend of the latter was weaker, which may be related to sampling period peaking in plant litter and root exudates that prefer *Actinobacteria* rather than the more oligotrophic *Acidobacteria* (Fierer et al., 2007). Furthermore, increases in the relative abundance of *Actinobacteria* may be due to the reduction of *Acidobacteira*, which might also be partly explained by these two groups share similar niches (Marco, 2008).

Like soil nutrients, bacterial communities were spatially distributed in the black soil zone in northeast China in accordance with soil pH and soil carbon content (Liu et al., 2014). In wetlands, complex soils, and sediments, water regimes were key factors determining bacterial community structure (Ligi et al., 2014); nitrate and C:N ratio were most dominant in shaping the archaeal community structure (Li et al., 2017). In the Antarctic terrestrial ecosystem, soil microbial nitrogen cycle was dramatically altered by temperature and nitrogen, especially warming was detrimental to the ammonia-oxidizing archaeal community (Jung et al., 2011). In our study, the selected environmental factors, Y value of spatial location, elevation, TP, and plant density drove the soil microbial taxa distribution in the karst forest soils (**Figure 5**). Moreover, variance partitioning showed that spatial factors better explained the total variation of soil microbial communities than environmental factors, which indicated that dispersal limitation was the primary driver of soil microbial spatial pattern in broadleaved forests in karst areas (Martiny et al., 2006). However, a large portion of the variation (76.8%) was unexplained in our study. This suggested that the spatial patterns of soil bacterial taxa in the karst forest soils were complex. The unexplained factors may include unmeasured environmental variables (e.g., soil moisture and temperature), inter- and intra-specific relationships of among microbial communities and among plant species, as well as plant-microorganism interactions.

## Conclusion

The dominant microbial phylum in the karst forest soils was *Proteobacteria* (34.51%), which was dominated by *Alpha-* and *Deltaproteobacteria*. *Actinobacteria* (30.73%) and *Acidobacteria* (12.24%) were other dominating phyla. We demonstrated that spatial patterns of the microbial taxa relative abundance at the phyla level could be detected and modeled at the study scale, which suggested that the microbial communities were spatially distributed in the karst forest soil of southwest China. Moreover, kriging maps displayed that no single spatial pattern was shared by all the main bacterial communities, which indicated that the patterns of microbial phyla in the karst forest soils were heterogeneous and complex. Furthermore, location, soil TP, elevation, and plant density were significantly correlated with main soil bacterial taxa in the karst forest. However, the total variation in soil microbial communities better explained by spatial factors than environmental variables. Moreover, a large portion of the variation was unexplained in our study. Our results suggested that dispersal limitation was the primary drivers of soil microbial distribution in broadleaved forest in karst areas, and environmental factors (e.g., soil porosity and temperature) may be taken into consideration.

## Author Contributions

WP, FZ, and QX were responsible for experiment design and writing guidance. MS and WP were responsible for experiment performance and paper writing. QP, HD, LC, and FZh were responsible for experimental data processing and analysis.

## Conflict of Interest Statement

The authors declare that the research was conducted in the absence of any commercial or financial relationships that could be construed as a potential conflict of interest.

## Footnotes

1 http://www.ctfs.si.edu
