# Supplementary material for: Spatial Patterns and Drivers of Microbial Taxa in a Karst Broadleaf Forest
Source: Front Microbiol. 2018 Jul 26;9:1691. doi: 10.3389/fmicb.2018.01691 (PMC6070632; doi:10.3389/fmicb.2018.01691)
Supplement: Supplementary file 1 [file Presentation_1.PDF]

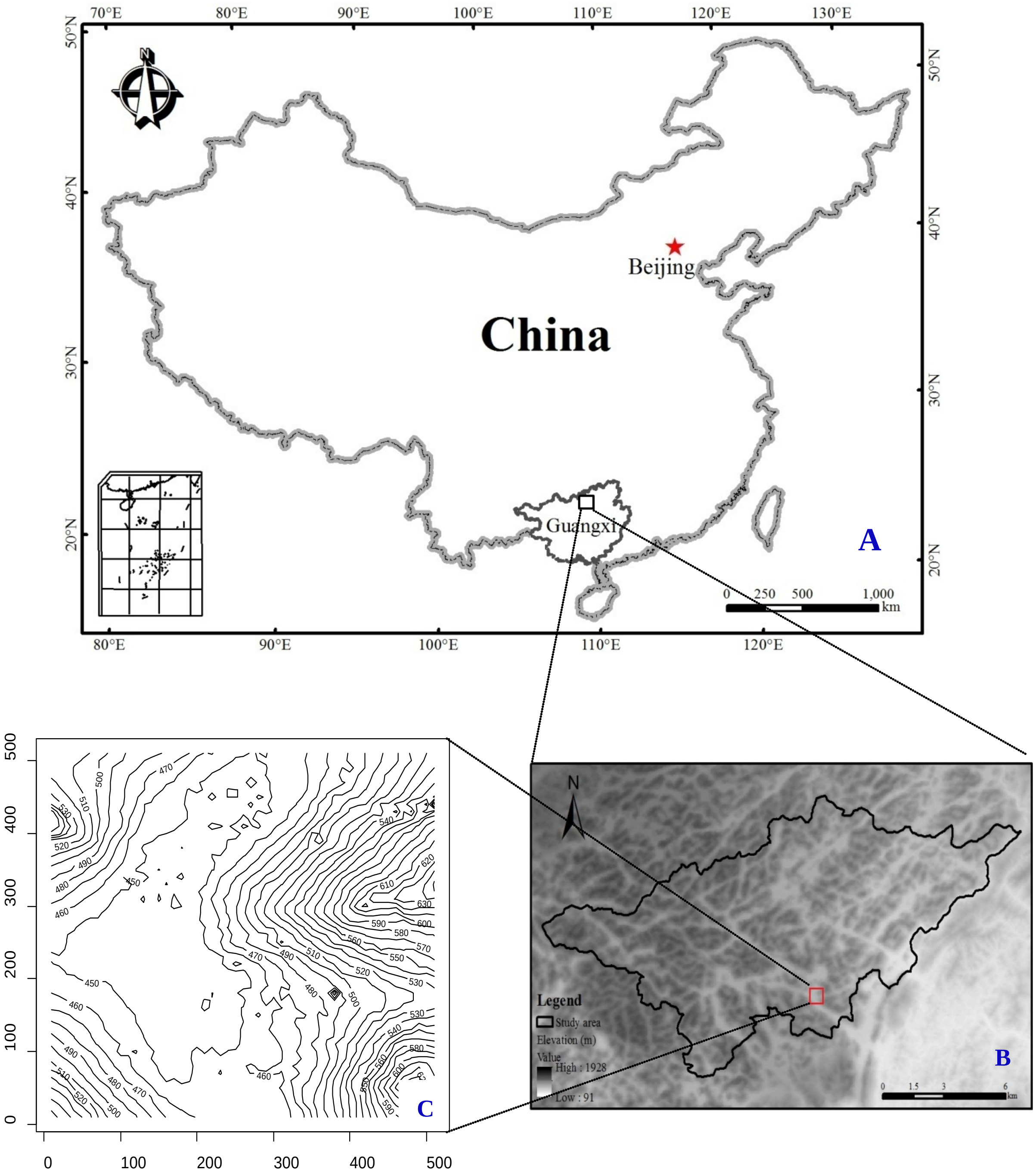

Fig.1S Location of the study area in Mulun National Nature Reserve, China. Maps generated using ArcGis 10.0.

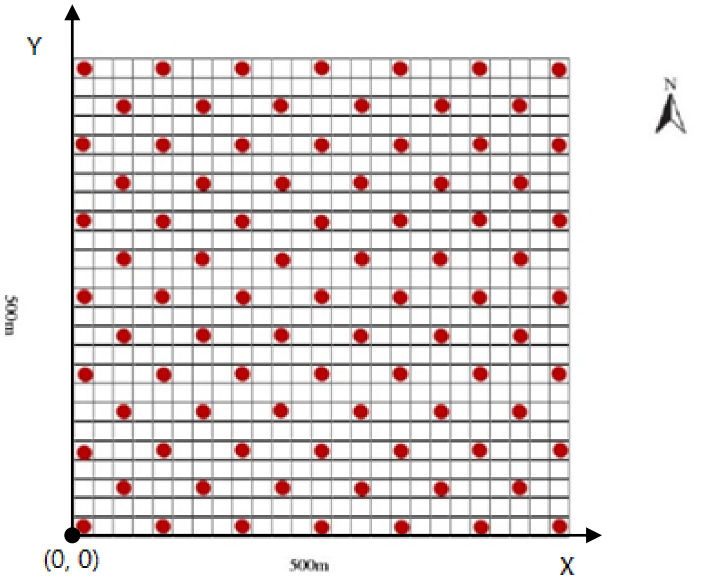

Fig.2S Soil sample collection design

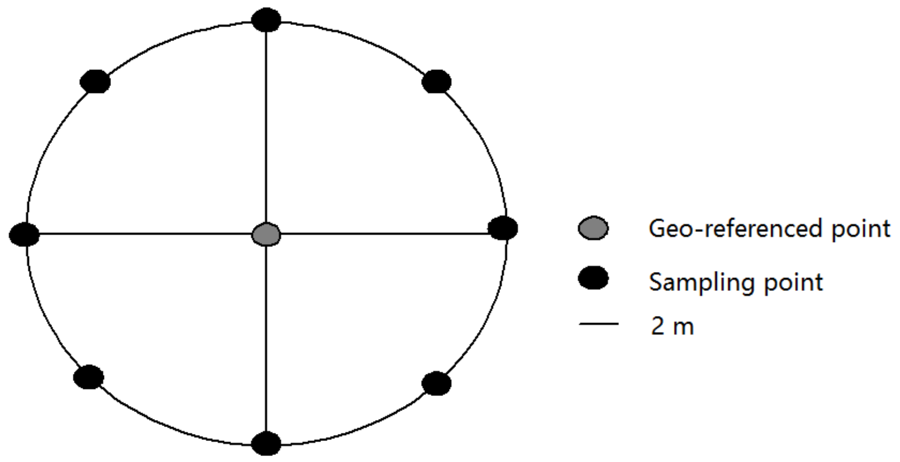

Fig.3S Scheme showing how sampling was performed. Eight soil samples were taken around a geo-referenced point and were mixed in order to constitute a composite sample representing that given point.

Fig.4S Relative abundances of the dominant bacteria at phylum and class taxonomic levels in the karst forest

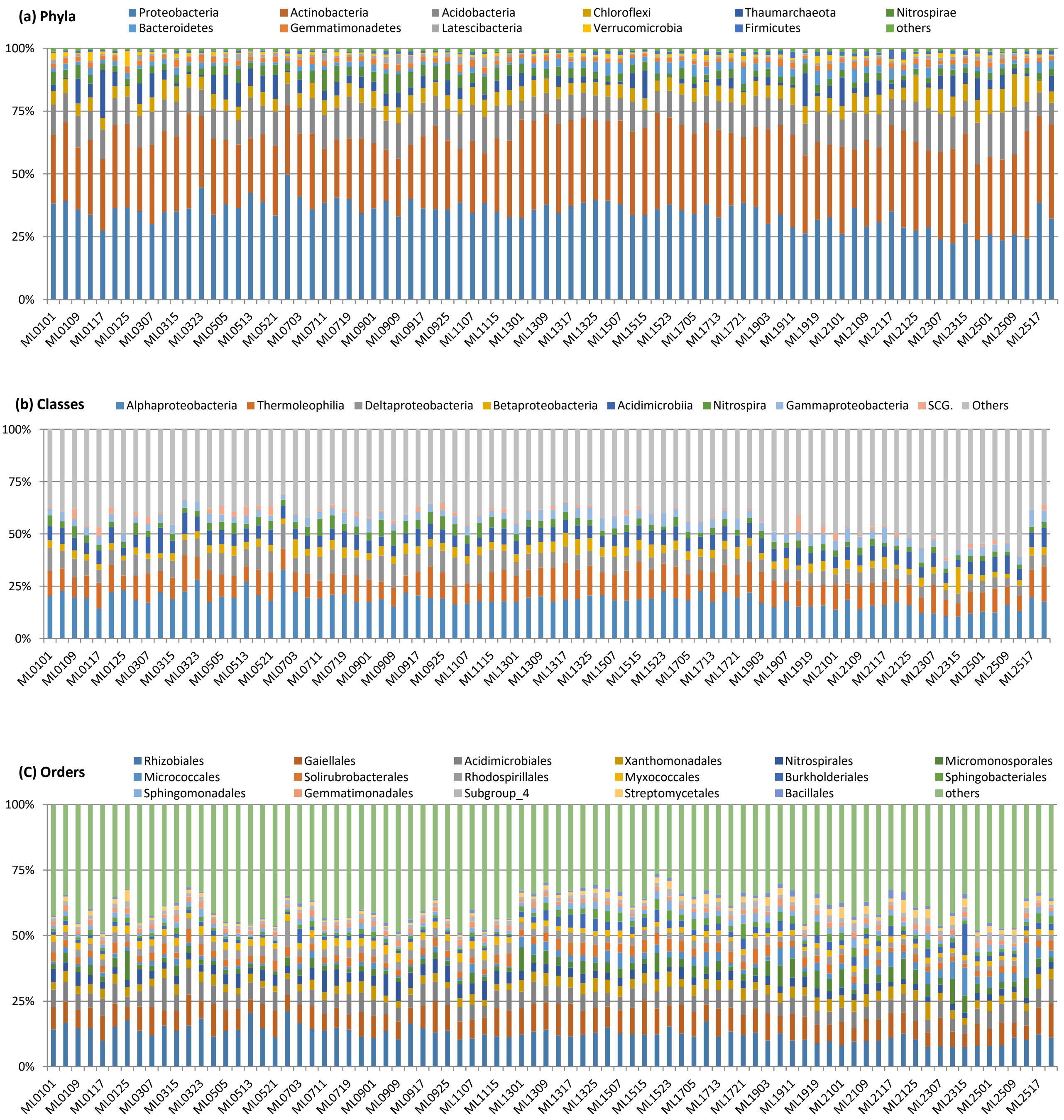

Table 1S Statistical description of the relative abundance of the main phyla in the karst forest

	N	Mean	SD	CV(%)	Skewness	Kurtosis	K-S Value	*P*
Actinobacteria	82	.307	.049	15.96	-.264	-.330	.556	.917
Alphaproteobacteria	82	.184	.004	20.11	0.666	2.667	0.874	.430
Acidobacteria	82	.122	.024	19.67	.491	.527	.960	.316
Deltaproteobacteria	82	.078	.002	28.21	0.654	-.092	1.018	.251
Chloroflexi	82	.060	.016	26.67	1.848	4.246	1.890	.002*
Thaumarchaeota	82	.042	.035	83.33	1.398	2.912	1.123	.160
Nitrospirae	82	.036	.014	38.89	1.373	1.753	1.610	.011*
Bacteroidetes	82	.024	.013	54.17	1.069	.987	1.091	.185
Gemmatimonadetes	82	.020	.005	25.00	.500	-.219	.911	.377
Latescibacteria	82	.011	.008	72.73	1.922	3.684	1.711	.006*
Verrucomicrobia	82	.010	.008	80.00	3.168	15.070	1.732	.005*
Firmicutes	82	.010	.006	60.00	1.875	5.626	1.209	.108

* indicates the data obey normal distribution after Cox-Box transformation.

Table 2S Semivariance analysis of spatial structure of relative abundance of the main phyla

	Model	C_0	Sill ($C+C_0$)	$C/(C+C_0)$	R^2	RSS	Rang (m)
Actinobacteria	Spherical	0.001383	0.002946	0.5305	0.972	2.734×10^{-8}	412.8
Alphaproteobacteria	Guassian	0.000192	0.000597	0.678	0.991	7.705×10^{-10}	368.9
Acidobacteria	Spherical	0.000291	0.000582	0.5000	0.933	1.769×10^{-9}	208.5
Deltaproteobacteria	Gaussian	0.00715	0.0025	0.714	0.992	1.848×10^{-8}	883.0
Chloroflexi	Spherical	0.0753	0.1946	0.6131	0.804	1.114×10^{-3}	210.1
Thaumarchaeota	Exponential	0.00004	0.00121	0.9669	0.004	4.301×10^{-8}	44.4
Nitrospirae	Exponential	0.000048	0.0002584	0.8142	0.977	2.414×10^{-10}	385.8
Bacteroidetes	Spherical	0.0000693	0.0001746	0.6031	0.977	1.060×10^{-10}	295.3
Gemmatimonadetes	Spherical	0.0000143	0.0000324	0.5586	0.894	7.607×10^{-11}	160.1
Latescibacteria	Spherical	3.52	11.50	0.6939	0.867	13	168.9
Verrucomicrobia	Exponential	0.008	0.413	0.9806	0.230	7.843×10^{-3}	84.3
Firmicutes	Exponential	0.0000286	0.0000575	0.5026	0.908	1.529×10^{-11}	710.9

RSS: residual sum of squares.

Table 3S Pearson's correlation coefficients among the environmental factors after conducting Benjamini-Hochberg procedure (n=82)

	X	Y	pH	SOC	TN	TP	TK	AN	AP	AK	S	Shannon	Simpson	Den	Sdepth	Rockcov	Elev	Slope
Y	-.051																	
pH	.100	.220																
SOC	.061	-.117	-.080															
TN	-.020	.019	-.133	.242														
TP	-.240	-.038	**-.329**[*]	.044	.243													
TK	-.260	-.072	-.084	.245	**.265**[*]	**.414**[*]												
AN	-.100	-.042	-.091	**.427**[*]	**.365**[*]	.085	.091											
AP	.037	-.227	-.178	.068	-.109	.254	.074	-.004										
AK	-.116	-.195	-.195	.238	**.348**[*]	.144	.206	**.423**[*]	.002									
S	**.408**[*]	.114	**.370**[*]	.048	.098	**-.301**[*]	-.080	-.104	**-.286**[*]	-.258								
Shannon	**.460**[*]	.101	**.322**[*]	.170	.236	**-.267**[*]	-.048	.114	-.224	-.009	**.897**[*]							
Simpson	**.444**[*]	.094	**.296**[*]	.178	.252	**-.272**[*]	-.052	.182	-.204	.099	**.789**[*]	**.966**[*]						
Den	.221	.107	**.346**[*]	-.078	-.201	-.259	-.199	**-.267**[*]	-.209	**-.375**[**]	**.564**[*]	**.315**[*]	**.249**					
Sdepth	-.241	.133	-.059	**-.296**[*]	**-.443**[*]	-.078	-.075	-.207	.004	-.255	**-.382**[*]	**-.547**[*]	**-.543**[*]	.081				
Rockcov	.177	-.177	.092	**.266**[*]	**.276**[*]	.109	.255	.182	-.141	.068	**.396**[*]	**.472**[*]	**.461**[*]	.026	**-.637**[*]			
Elev	**.587**[*]	-.040	**.329**[*]	.121	-.121	**-.410**[*]	**-.274**[*]	-.150	-.180	-.194	**.692**[*]	**.651**[*]	**.596**[*]	**.583**[*]	**-.303**[*]	**.289**[*]		
Slope	**.449**[*]	-.026	**.370**[*]	.210	.162	**-.310**[*]	-.150	.032	**-.278**[*]	-.145	**.813**[*]	**.788**[*]	**.729**[*]	**.560**[*]	**-.428**[*]	**.451**[*]	**.778**[*]	
Aspect	**.641**[*]	.121	.039	.009	.002	-.184	**-.273**[*]	-.023	.089	-.078	.228	.259	.249	.130	-.164	.078	.233	.202

[*] indicates that correlations are significant at Q-value <0.05 after p-value adjusting by Benjamini-Hochberg procedure. X and Y represent the location of the plots; soil pH value (pH), soil organic carbon (SOC), total nitrogen (TN), total phosphorus (TP), total potassium (TK), available nitrogen (AN), available phosphorus (AP), available potassium (AK); Richness index (S), Shannon index (Shannon), Simpson index (Simpson), plant density (Den); Soil depth (Sdepth), rock outcrop coverage (Rockcov), elevation (Elev), slope aspect (Aspect).
